# Filler Effects on H_2_ Diffusion Behavior in Nitrile Butadiene Rubber Blended with Carbon Black and Silica Fillers of Different Concentrations

**DOI:** 10.3390/polym14040700

**Published:** 2022-02-11

**Authors:** Jae Kap Jung, Chang Hoon Lee, Min Seok Son, Ji Hun Lee, Un Bong Baek, Ki Soo Chung, Myung Chan Choi, Jong Woo Bae

**Affiliations:** 1 Hydrogen Energy Materials Research Center, Korea Research Institute of Standards and Science, Daejeon 34113, Korea; ljh93@kriss.re.kr (J.H.L.); ubbaek@kriss.re.kr (U.B.B.); 2 Department of Biochemical and Polymer Engineering, Chosun University, Gwangju 61452, Korea; artphys63@gmail.com (C.H.L.); sonms@naver.com (M.S.S.); 3 Department of Physics and Research Institute of Natural Science, Gyeongsang National University, Jinju 52828, Korea; chungks@gnu.ac.kr; 4 Rubber Research Division, Korea Institute of Footwear & Leather Technology, Busan 47154, Korea; mcchoi@kiflt.re.kr (M.C.C.); jwbae@kiflt.re.kr (J.W.B.)

**Keywords:** nitrile butadiene rubber (NBR), carbon black filler, silica filler, volumetric analysis, hydrogen uptake, diffusion, permeability

## Abstract

Filler effects on H_2_ diffusion in nitrile butadiene rubbers (NBRs) blended with carbon black and silica fillers of different concentrations are first investigated by employing a volumetric analysis. Total uptake, solubility, and diffusivity of hydrogen for ten filled-NBR, including neat NBR, are determined in an exposed pressure range of 1.3 MPa~92.6 MPa. Filler dependence on hydrogen uptake and diffusion is distinctly observed in the NBRs blended with high abrasion furnace (HAF) carbon black (CB) fillers compared to NBRs blended with medium thermal furnace (MT) CB and silica filler, which is related to the specific surface area of carbon black and interface structure. The HAF CB filled-NBR follows dual sorption behavior combined with Henry’s law and the Langmuir model, responsible for two contributions of solubility from polymer and filler. However, a single gas sorption behavior coming from the polymer is observed satisfying Henry’s law up to 92.6 MPa for NBR blended with MT CB filled-NBR and silica filled-NBR. Diffusion demonstrates Knudsen and bulk diffusion behavior below and above, respectively, at certain pressures. With increasing pressure, the filler effect on diffusion is reduced, and diffusivity converges to a value. The correlation observed between diffusivity and filler content (or crosslink density) is discussed.

## 1. Introduction

Nitrile butadiene rubber (NBR) is a commonly utilized elastomer in the automotive, aeronautical and nuclear industries [1,2]. NBR is widely used in the seal industry for applications of O-rings, gaskets, seals for oil and gas, hydraulic seals and self-sealing fuel tanks [3,4]. Although NBR retains outstanding properties, enhancement fillers are necessarily added to NBR to attain appropriate properties for specific applications, such as low gas transport under high pressure. The reinforcement of elastomers improves physical properties such as tear strength, tensile strength, hardness, abrasion resistance and thermal properties. A wide variety of particulate fillers are used in the rubber polymer industry for various purposes, of which the most important are reinforcement, reduction in material costs, and improvements in processing [5,6]. Physical properties such as volume swelling, density, and the glass transition temperature of rubber vulcanizates are also improved by strengthening with fillers such as carbon black (CB) [7] and silica [8].

CB is one of the most widely used conductive nanoparticles in industrial applications and is considered a suitable candidate because of its low cost, good dispersion ability, and, especially, synergistic effect with carbon nanotubes [9,10]. When CB is compounded with rubbers, the tensile strength, tear strength, modulus and abrasion resistance are improved [10,11]. Therefore, CB has been extensively exploited in numerous rubber engineering products [12]. However, silica possesses the unique combination of tear strength, abrasion resistance, aging resistance and adhesion properties [13,14]. Synthetic silica is currently extensively used to improve physical and mechanical parameters, such as tensile strength and elongation of silicone rubber vulcanizates [12].

There are numerous studies on permeability in rubbery polymers. Some additives used as fillers in various polymers may affect the permeability [15,16,17,18]. The comparable effect of these fillers in reducing permeability and prolonging the diffusion path for polymers was investigated theoretically and experimentally [19,20]. 

The potential of inorganic fillers with different shapes and sizes to reduce permeability was systematically approached by employing a hydrogenated nitrile rubber (HNBR). The corresponding fillers, such as carbon black (N550) and precipitated silica, were incorporated at loadings up to 30 phr (parts per 100 parts of rubber). In the presence of 30 phr CB and precipitated silica, the diffusion coefficient decreases by 14–15% for both types of filler. Regarding the permeation coefficient, the incorporation of silica into HNBR leads to a reduction of 20%, whereas CB does not greatly affect the permeation rate of the composite [21]. It was reported in thermal desorption analysis–gas chromatography that the addition of CB filler of 50 phr in NBR composites was responsible for increased hydrogen contents and decreased diffusivity by adsorbed hydrogen, as a result of the trapping of hydrogen by the CB. Especially, the diffusion behavior in NBR displays dual mode behaviors with fast and slow components [22].

Under these research motivations, it is necessary to conduct an investigation into the effects of fillers on these related properties in rubber composites with the aid of an appropriate permeation measurement technique. Recently, we developed an effective and simple measurement technique for characterizing hydrogen gas permeation properties in rubber polymers [23,24]. The technique combines a volumetric analysis measurement of emitted H_2_ gas using a graduated cylinder and diffusion analysis program. The volumetric analysis measurement is a very appropriate and stable method to determine the permeation properties, regardless of specimen shape/dimension and gas species. In addition, diffusion analysis program for simulating hydrogen transport property is upgraded for use of various gases (He, N_2_, O_2_ and Ar), different shapes (cylinder, sphere and sheet) specimen at both modes of emission and remaining content of gas. For the verification of the developed technique, we have already verified the volumetric analysis technique (VAT) in previous studies [23] by comparing the results obtained by VAT with those by different methods, such as gas chromatography by thermal desorption analysis, gravimetric measurement by electronic balance for same samples. The results were found to be consistent with each other within uncertainty.

Based on this research and accumulated techniques, the present investigations are concerned with studies of the hydrogen permeation properties of blended-NBR mixed with CB filler of different particle sizes and silica filler including neat NBR. The effect of filler loading on hydrogen permeation was studied in an attempt to understand the corresponding sorption kinetics/mechanism and filler-induced effects. This work also presented precise data on the gas permeability characteristics of polymer blended materials used for hydrogen gas sealing materials. The hydrogen uptake, solubility and diffusivity of the NBR polymer composites blended with three kinds of fillers were systematically first investigated as a function of the exposed pressure, filler content and crosslink density. From the investigations for filler effects, we could characterize quantitatively H_2_ sorption phenomena, that is, single sorption or dual sorption behaviors depending on fillers type and its spices in filled-NBR composites. Moreover, the correlation between diffusivity and crosslink density is derived. 

## 2. Materials and Methods

### 2.1. Sample Composition

The detailed composition and growing method for the specimens have already been described in previous literature [25]. KNB 35 L (Kumho NBR) with an acrylonitrile content of 34 wt %, produced by the Kumho petrochemical group, is used as the main component for neat NBR rubber. The compound recipe for the chemical composition for NBR specimens with CB and silica fillers is given in Table 1 and Table 2, respectively, including one neat NBR without any added filler, six samples with CB and three samples with silica filler. In this study, we employed two types of CB prepared using a high abrasion furnace (HAF, N330) and a medium thermal furnace (MT, N990) by Orion Engineer Carbon, which have particle sizes of 28–36 nm and 250–350 nm, respectively. The specific surface areas of HAF and MT are 76 m^2^/g and 8 m^2^/g, respectively. Silica was produced by Zeosil^®^ 175 from Solvay, with a specific surface area of 175 m^2^/g. The vulcanizates were filled with 20 phr, 40 phr and 60 phr (parts per 100 parts of rubber). For simplicity, the NBR blends mixed with fillers are named NBR-Hx, NBR-My, and NBR-Sz, where x, y, and z indicate the phr content for HAF, MT and silica, respectively. For example, NBR-S40 is NBR filled with silica of 40 phr.

Two-stage mixing is employed using an internal mixer with two Banbury rotors and two open roll mills of eight inches to prepare NBR composites. The first stage mixing (masterbatch) was compounding NBR rubber, reinforcing fillers such as CB and precipitated silica, and processing aids such as ZnO and stearic acid with an internal mixer (3 L kneader, Moriyama Co., Sanda, Japan). The filling factor was fixed to 0.8, and the starting operation temperature of the kneader was set to 80 °C. The rotor speed was set to 30 rpm. NBR rubber was added to a 3-L kneader and masticated for 3 min. Then, the reinforcing filler and the processing aids were incorporated for 10 min. In the second stage of mixing, open roll mills were used to add the curing agents and accelerating agents into the masterbatch composite. The mixer was set to a nip opening of 3 mm between the rolls. The master batch was added to the roller and mixed for 1 min. Sulfur and TBBS were then added and mixed into the batch, which took approximately 2 min. The mixer nip was opened, and then the finished batch was cut into sheets. The mixing time was kept uniform for all composites. Vulcanizate sheets of the composites with a thickness of 3 mm for diffusion measurement were prepared by compression molding in a hydraulic press at 150 ℃ based on the optimum cure time obtained from an oscillating disk rheometer. The dimension of the cylindrical shaped rubber specimen used is ~12 mm for diameter and ~3 mm for thickness. The surface morphologies on the cryo-fractured cross-section for polymer specimen have been investigated with a Field Emission Scanning Electron Microscopy (FE-SEM, Hitachi S4800, Tokyo, Japan) in the mixing mode together with lower and upper detection at 15 kV and 10 μA at room temperature. Compared to SEM, the FE-SEM provides a high-resolution image which is of the advantage for polymer characterization.

### 2.2. Exposure Condition to Hydrogen

High-pressure chamber and purge conditions are described in the preceding literature [23,24]. An SUS 316 chamber was used for gas exposure to high pressure at room temperature. The chamber was purged three times with the corresponding gas at 1~5 MPa depending on the exposed pressure before gas exposure. We exposed the gas for 30 h to the specimen in the pressure ranging from 1.5 MPa to 92.6 MPa. Hydrogen gas charging for 30 h was enough to attain the equilibrium state for gas sorption. After exposure to gas, the valve was opened, and the gas in the chamber was released. After decompression, the elapsed time was recorded from the moment at which the high pressure in the chamber was reduced to atmospheric pressure when the time was set to zero, *t* = 0. Since the specimen was loaded in the graduated cylinder after decompression, approximately 5~15 min were required to start the measurement. The gas content emitted for the inevitable time lag could be measured by offset determination via a diffusion analysis program.

## 3. Measurement Method and Data Analysis

### 3.1. Volumetric Measurement of Emitted Hydrogen

**Figure 1:**
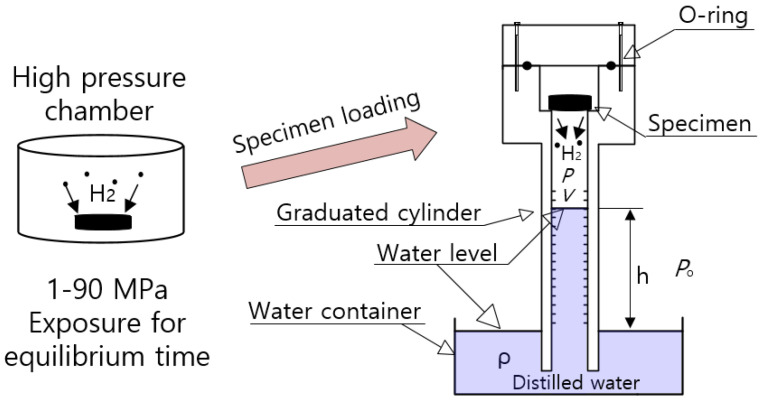
Configuration of the volumetric measurement system using a graduated cylinder after high-pressure exposure for 30 h and subsequent decompression. The time lag due to specimen transfer (sample loading) amounts to 5~15 min. The blue color indicates distilled water.

Figure 1 shows the volumetric measurement system with a graduated cylinder used to measure the released hydrogen gas. After exposure to the high-pressure chamber for 30 h and subsequent decompression, the specimen is loaded into the gas space of the top side in a graduated cylinder. The hydrogen gas emitted from the specimen lowers the water level of the graduated cylinder. By reading the graduations on a standing graduated cylinder immersed partially in a water container, we could measure the amount of hydrogen gas released from the specimen. Descriptions of the volumetric method were also found in previous literature [23,24].

The pressure of gas (P) inside the graduated cylinder is expressed as:

(1) $$P=P_{o}-\rho gh$$

$P_{o}$ is the atmospheric pressure outside the cylinder, ρ  is the density of distilled water in the water container, and *g* is gravity. h is the height of the water level inside the graduated cylinder measured from the water levels in the water container. V is the gas volume inside the graduated cylinder filled with hydrogen gas and air, as shown in Figure 1. The gas inside the cylinder is governed by the ideal gas equation, *PV* = *nRT*, and R is the gas constant of 8.20544 × 10^−5^ m^3^·atm/(mol·K).

The total number of moles (n) of gas inside the cylinder is expressed as follows [23,24]:

(2) $$n=n_{0}+\Delta n=\frac{(P_{o}-\rho gh)V}{RT}$$

*T* is the temperature of gas occupied in the cylinder in Figure 1. $n_{0}$ is the initial mole number of the air already in the cylinder before gas emission. The temperature and pressure measured near the sample are applied for the calculation of gas uptake. The gas released from the specimen after decompression lowers the water level of the cylinder. Thus, the increased number of moles (Δn) of gas emitted in the cylinder after decompression is obtained by measuring the increase in volume (ΔV) in the graduated cylinder, i.e., lowering of the water level as follows:

(3) $$\Delta n=\frac{(P_{o}-\rho gh)\Delta V}{RT}$$

The increased number of moles in the cylinder is converted to the mass concentration [C(t)] of gas emitted from the rubber sample as follows:

(4) $$C\left(t\right)\left[\mathrm{wt}\cdot \mathrm{ppm}\right]=\Delta n\left[\mathrm{mol}\right]\times \frac{m_{H2}\;\left[\frac{g}{\mathrm{mol}}\right]}{m_{\mathrm{sample}}\left[g\right]}\times 10^{6}$$

$m_{H2}$ [g/mol] is the molar mass of H_2_ gas, 2.016 g/mol. $m_{\mathrm{sample}}$ is the mass of the specimen. By measuring the change in the water level, an increased mole number is obtained, and then the mass concentration of the emitted gas is transformed by Equation (4). Therefore, the time-dependent mass concentration of the released gas from the specimen is obtained by measuring ΔV versus elapsed time after decompression.

### 3.2. Diffusion Analysis Program

The adsorption of gas at high pressure causes the emission of gas dissolved in the polymer after decompression to atmospheric pressure. Assuming that the adsorption and desorption of gas is a diffusion-controlled process, the emitted gas concentration $C_{E}\left(t\right)$ in the desorption process is expressed as [26,27]

(5) $$C_{E}\left(t\right)/C_{\infty }=1-\frac{32}{\pi^{2}}\times \left[\overset{\infty }{\underset{n=0}{\sum }}\frac{exp\left\{\frac{-{\left(2n+1\right)}^{2}\pi^{2}Dt}{l^{2}}\right\}}{{\left(2n+1\right)}^{2}}\right]\times \left[\overset{\infty }{\underset{n=1}{\sum }}\frac{exp\left\{-\frac{D\beta_{n}^{2}t}{\rho^{2}}\right\}}{\beta_{n}^{2}}\right]$$

Equation (5) is the solution to Fick’s second diffusion law for a cylindrical rubber specimen under the boundary condition with an initially constant uniform gas concentration and constant concentration at the cylindrical surface. $C_{\infty }$ is the saturated hydrogen mass at an infinitely long time, i.e., the total emitted mass concentration or hydrogen uptake in the adsorption process. *D* is the diffusion coefficient. l is the thickness of the sample, ρ is the radius, and $\beta_{n}$ is the root of the zero-order Bessel function.

**Figure 2:**
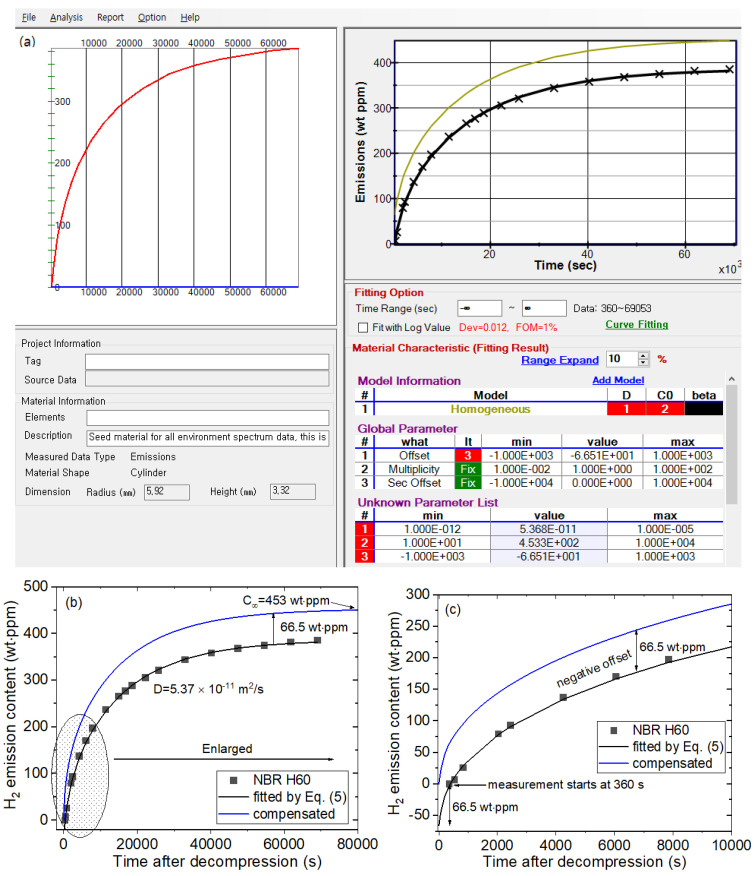
(**a**) Application example of the diffusion analysis program to obtain the hydrogen uptake and diffusion coefficient for NBR H60 exposed to 8.9 MPa. (**b**) The redrawn diffusion analysis result and (**c**) the enlargement of the ellipse part in (**b**).

To analyze the mass concentration data using Equation (5), a developed diffusion analysis program to calculate *D* and $C_{\infty }$, based on a least-squares regression and Nelder–Mead simplex optimization algorithm [23,24,28], is applied. Figure 2 shows the representative analysis result of the diffusion analysis program for NBR H60 rubber exposed to 8.9 MPa. In the bottom left of Figure 2a, the radius and height (thickness) of the specimen with a cylindrical shape are entered. As shown in the right frame of Figure 2a, the × symbol and black line indicate the experimental data and line fitted with Equation (5), respectively. *D* and $C_{\infty }$ are obtained by substituting the hydrogen emission content at each time into Equation (5) and optimizing each parameter by the least-squares method. The values *D* = 5.37 × 10^−11^ m^2^/s and $C_{\infty }$ = 453 wt·ppm with a negative offset of −66.5 wt·ppm (yellow line) are acquired, as shown in the unknown parameter list of Figure 2a. 

Meanwhile, the hydrogen emission contents are missing during the time lag between decompression and the start of measurement. Thus, the missed contents are restored in the following manner. Figure 2b is redrawn results of Figure 2a. Figure 2c is an enlargement of the ellipse part in Figure 2b. As the measurement started at 360 s after decompression due to the time lag, the emission value at *t* = 360 s is 0. However, the emitted hydrogen quantity should be 0 when *t* = 0. Therefore, we can compensate for the missing value between *t* = 0 and *t* = 360 s by upshifting with an offset of 66.5 wt·ppm (blue line) corresponding to a negative y value at t = 0 on the fitted black line in Figure 2c. This value, indicated as the difference between the blue line and black line in Figure 2c, is obtained by extrapolating the fitted black line satisfying the data according to Equation (5) with the analysis program. This offset value should be included to precisely determine *C*_∞_. Thus, the final hydrogen uptake including the offset value is *C*_∞_ = 453 wt·ppm.

## 4. Results and Discussion

### 4.1. Filler Effect on Pressure-Dependent H_2_ Solubility

**Figure 3:**
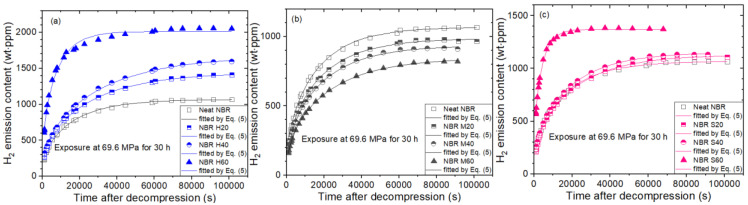
Time-varying H_2_ emission content for (**a**) NBR H series, (**b**) NBR M series and (**c**) NBR S series with neat NBR after hydrogen exposure of 69.6 MPa for 30 h and decompression. The three same NBR neat results are shown to each figure for comparison with filled-NBR.

We measured the hydrogen emission content versus the elapsed time after decompression in the pressure ranging from 1.3 MPa to 92.6 MPa for ten cylindrical-shaped NBR composites filled with CB and silica, including neat NBR. Figure 3 shows the representative H_2_ emission results for ten NBR composites after hydrogen exposure of 69.6 MPa. Figure 3a depicts the time-varying H_2_ emission content for one neat NBR and three NBR composites with HAF CB fillers of 20 phr (NBR H2O), 40 phr (NBR H40) and 60 phr (NBR H60). The hydrogen uptake and diffusion rate increase with increasing HAF CB filler content. Meanwhile, the filler effect on NBR composites with MT CB, as shown in Figure 3b, exhibits different behavior compared with Figure 3a. The hydrogen uptake decreases with increasing MT CB filler content, whereas the diffusion rate for neat NBR is almost the same as those for NBR composites with MT CB filler. The different influences are attributed to the different particle sizes and specific surface areas between the two kinds of CB fillers.

Meanwhile, the variation in H_2_ emission content versus time by silica filler content does not show an appreciable change in Figure 3c, except for NBR S60. The rapid increase in the diffusion coefficient for NBR H60 in Figure 3a and NBR S60 in Figure 3c may be responsible for the formation of hydrogen channels by filler percolation after exposure to 69.6 MPa, which was later confirmed by scanning electron microscopy (SEM).

**Figure 4:**
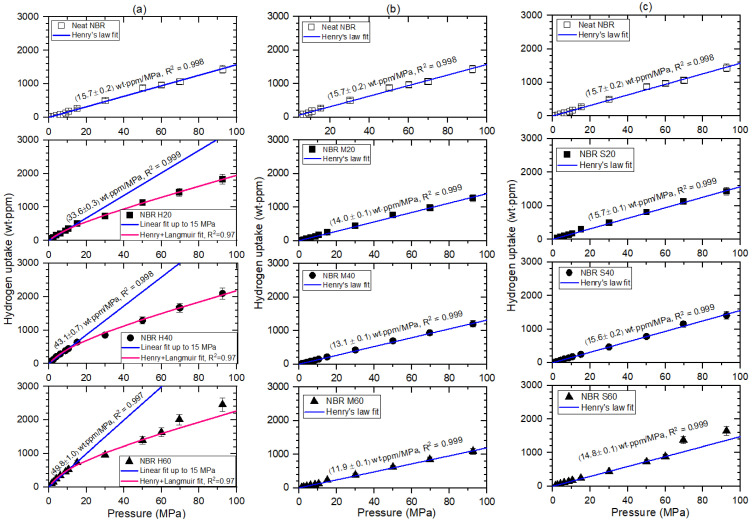
Hydrogen uptake ($C_{\infty }$) versus exposed pressure for (**a**) NBR H series, (**b**) NBR M series and (**c**) NBR silica series. The blue lines for all specimens indicated as the slope of hydrogen uptake with respect to pressure, with a squared correlation coefficient R2, is the result of Henry’s law fit. The last two points for NBR S60 in (**c**) are excluded from Henry’s law fit because of deviation. The pink lines in (**a**) are the dual sorption fits by both Henry’s law and the Langmuir model. The last two points for NBR H60 in (**a**) are excluded from dual sorption fit because of deviation. Three identical neat NBR results shown at the top of each figure are inserted for comparison with filled-NBR composites.

Figure 4 illustrates the hydrogen uptake versus pressure for NBR composites blended with fillers including neat NBR. Figure 4a–c shows the pressure dependence of the hydrogen uptake NBR composites blended with HAF CB filler, MT CB filler and silica filler, respectively. The three same neat NBR results are included at the top of each figure for comparison with filled-NBR composites. As shown in Figure 4, the hydrogen uptake for neat NBR, MT CB filled-NBR and silica filled-NBR is proportional to the pressure up to 92.6 MPa satisfying Henry’s law [29], except for HAF CB filled-NBR, as follows:

(6) $$c_{\infty }=kP$$

where *k* is Henry’s constant and P is the pressure. Henry’s law fit results are represented by the hydrogen uptake slope for pressure indicated by the blue lines in Figure 4, implying that hydrogen does not dissociate and penetrates into the polymer as a hydrogen molecule.

Meanwhile, the second, third and fourth components from the top of Figure 4a correspond to the NBR composites blended with HAF CBs of 20 phr (NBR H20), 40 phr (NBR H40) and 60 phr (NBR H60), respectively, and display sorption content satisfying Henry’s law up to 15 MPa. The hydrogen uptake slope up to this pressure range is also indicated by the blue lines.

The hydrogen solubility (*S*) is acquired from the *C*_∞_ slope with respect to pressure by the following relation.

(7) $$S\left[\frac{\mathrm{mol}}{m^{3}\cdot \mathrm{MPa}}\right]=\frac{C_{\infty }\;\mathrm{slope}\;\left[\frac{\mathrm{wt}\cdot \mathrm{ppm}}{\mathrm{MPa}}\right]\times 10^{6}\times d\left[\frac{g}{m^{3}}\right]}{m_{H_{2}}\left[\frac{g}{\mathrm{mol}}\right]}$$

where *m*_H2_ is the molar mass of hydrogen, *m*_H2_(g/mol) = 2.016 g/mol, and *d* is the density of the NBR composites. Using Equation (7), the solubility of hydrogen for neat NBR and filled-NBR is determined as follows:

For neat NBR: *S* = (8.64 ± 0.69) mol/(m^3^ MPa)

For NBR H20: *S* = (19.2 ± 1.5) mol/(m^3^ MPa)

For NBR H40: *S* = (24.6 ± 2.0) mol/(m^3^ MPa)

For NBR H60: *S* = (28.5 ± 2.3) mol/(m^3^ MPa)

For NBR M20: *S* = (8.01 ± 0.64) mol/(m^3^ MPa)

For NBR M40: *S* = (7.49 ± 0.60) mol/(m^3^ MPa)

For NBR M60: *S* = (6.80 ± 0.54) mol/(m^3^ MPa)

For NBR S20: *S* = (8.97 ± 0.72) mol/(m^3^ MPa)

For NBR S40: *S* = (8.91 ± 0.71) mol/(m^3^ MPa)

For NBR S60: *S* = (8.46 ± 0.68) mol/(m^3^ MPa)

However, the hydrogen uptake for HAF CB-filled NBR composites deviates from Henry’s law at pressures above 15 MPa, which is attributed to adsorbed hydrogen at the surface of the HAF CB filter. Thus, the dual sorption behaviors for covering the overall pressure range up to 92.6 MPa can be introduced as follows.

(8) $$c_{\infty }=kP+\frac{abP}{1+bP}$$

The second term presents the Langmuir model [30], where a is the maximum adsorption quantity and b is the adsorption equilibrium constant. The dual sorption fit result according to Equation (8) is indicated by three pink lines in Figure 4a, as follows:

For NBR H20: *a* = (431 ± 47) wt∙ppm and *b* = (0.0691 ± 0.0114) MPa^−1^

with *k* = (15.7 ± 0.2) wt·ppm/MPa

For NBR H40: *a* = (705±77) wt∙ppm and *b* = (0.0624 ± 0.0095) MPa^−1^

with *k* = (15.7 ± 0.2) wt·ppm/MPa

For NBR H60: *a* = (782±86) wt∙ppm and *b* = (0.0799 ± 0.0129) MPa^−1^

with *k* = (15.7 ± 0.2) wt·ppm/MPa

where Henry’s law fit for three HAF CB filled-NBRs is assumed to be the same as the Henry’s law fit of neat NBR. The fitted result indicates that the maximum adsorption hydrogen quantity increases with increasing filler content. As shown in Figure 4a,c, the deviations from dual behavior above 70 MPa for NBR H60 and from Henry’s law above 70 MPa for NBR S60 indicate an abrupt increase in hydrogen sorption, which may be caused by the formation of hydrogen path channels at higher pressures. The related description will be discussed with SEM images in the following section.

**Figure 5:**
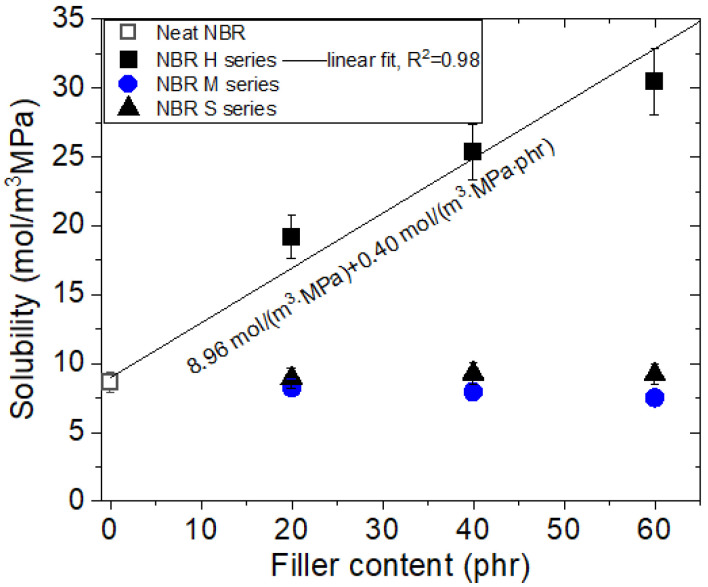
Variation in solubility versus different filler species and their content.

Figure 5 depicts the filler variation of solubility obtained just before in filled-NBR composites including neat NBR. The solubility of the HAF CB-filled NBR composite increases linearly with increasing HAF CB filler content, with a squared correlation coefficient R^2^ = 0.98. The intersection on the *y*-axis and slope of the linear fit correspond to 8.96 mol/(m^3^·MPa) and 0.40 mol/(m^3^·MPa·phr), respectively, implying that the hydrogen solubility comes from two contributions, i.e., neat polymer itself and HAF CB filler. The contribution from the parent polymer has a fixed predetermined value, while the contribution quantity from the HAF CB filler is proportional to the filler content.

However, the solubility for NBR composites with MT CB filler and silica filler was found to be constant irrespective of filler content and was nearly equal to the solubility for neat NBR, indicating that most hydrogen in blended NBR composites with MT CB and silica is absorbed into the polymer network itself and not adsorbed by the filler surface. The difference in CB filler effects may be attributed to the specific surface area of the CB filler. NBR composites with HAF CB filler of smaller filler size and ten times larger specific surface area than those for MT CB filler effectively contribute to the increase of solubility by filler.

The solubility values of silica-filled NBR are nearly equal to the solubility values of neat NBR, although the specific surface area of silica is larger than the specific surface area of CB filler, indicating that CB filler and silica differ in view of the interfacial structure. Therefore, the hydrogen solubility of filled-rubbers is inferred to be influenced not only by the surface area of fillers but also by the interface structure between the filler and polymer matrix.

The sorption phenomenon of polymers has been described in two different ways, whether the polymer is in the glass phase or not [31,32]. Above the glass transition temperature (T_g_), i.e., in the rubbery phase, sorbate sorption by the sorbent polymer is dominated by the absorption process, satisfying Henry’s law expressed as the first term of Equation (8). Here, the main and side chains of the polymer are assumed to fluctuate continuously by thermal energy, and thus, a void or pore structure needed for sorption is not kept constant but relaxed quickly. For below T_g_, i.e., in the glass phase, sorbate sorption is known to proceed by Henry’s absorption and Langmuir’s adsorption expressed as the 2nd term of Equation (8), leading to a dual sorption mechanism because the glass phase has a relaxed region and a robust void structure. Henry’s absorption is attributed to the relaxed region, and Langmuir’s adsorption is due to the robust void structure.

The NBR composite system is, in fact, at the rubbery phase at room temperature. Therefore, the dual mode sorption is not possible in the NBR composite. However, our experimental data show dual mode sorption for only HAF CB-filled NBR due to the existence of porous HAF CB filler. For the case of our NBR composite system, the NBR matrix is in the rubbery phase, while the porous filler has many pores. Thus, H_2_ molecules can be absorbed by rubbery NBR and simultaneously can be adsorbed by the porous filler, leading to dual mode sorption similar to the glass phase. In other words, the porous HAF CB filler in the NBR composite corresponds to the robust void structure in the glass phase. The solubility result in HAF CB-filled NBR, as shown in Figure 5, also supports the dual sorption behavior.

### 4.2. Filler Effect on Pressure-Dependent H_2_ Diffusivity

**Figure 6:**
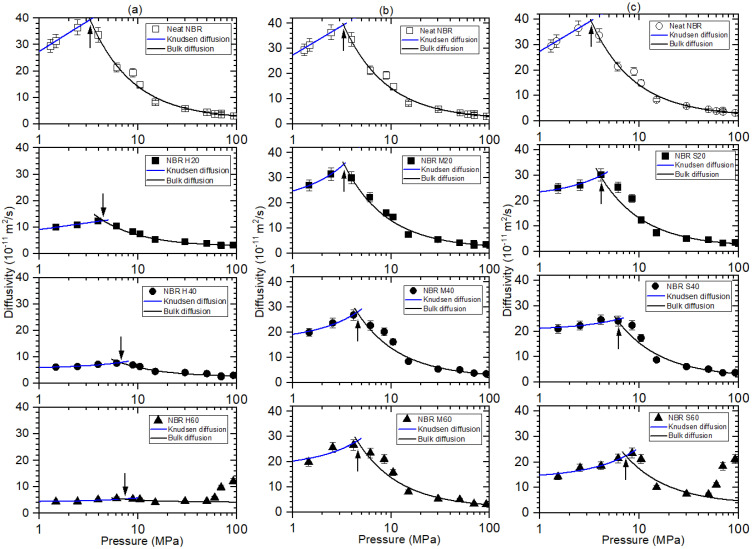
Hydrogen diffusivity (D) versus exposed pressure for (**a**) NBR H series, (**b**) NBR M series and (**c**) NBR S series. The blue line indicates that the slope of hydrogen uptake proportional to pressure is a Knudsen diffusion fit. The black line is the result of bulk diffusion. The last three points for NBR H60 and S60 are excluded from bulk diffusion fitting because of deviation. The arrow positions for (**a**–**c**) shift to the high-pressure side with increasing filler content. Three identical graphs for neat NBR results shown at the top of each figure are inserted for comparison with filled-NBR composites.

The hydrogen diffusivity in neat NBR and filled-NBR composites versus pressure is represented in Figure 6. As shown in Figure 6a–c, the pressure-dependent diffusion could be divided into two contributions at peaks indicated by arrows. The two contributions correspond to Knudsen diffusion for low pressure and bulk diffusion for high pressure. The pressure-dependent behavior on diffusivity was interpreted by the result of the combination of Knudsen diffusion below 3~7 MPa and bulk diffusion above the pressures, which was observed and analyzed by fractal theory in other studies [27,33]. The Knudsen diffusion gradually increases with increasing pressure. Knudsen diffusion below the pressures normally occurs for the case with a large mean free path of diffusing gas molecules or its low gas density. The Knudsen diffusion coefficient ($D_{K,\;pm}$) in porous media can be expressed as [34]:

(9) $$D_{K,pm}=\frac{\emptyset }{\tau }D_{K}=\frac{\emptyset }{\tau }\frac{d_{c}}{3}\upsilon$$

where ∅ is the pressure-dependent porosity, τ  is the tortuosity caused by introducing the filler, $d_{c}$ is the pore diameter, and υ is the average molecular velocity derived from the kinetic theory of gases.

Meanwhile, the bulk diffusion coefficients above 3.5 MPa for neat NBR, above a critical pressure of 3 MPa~7.0 MPa for filled-NBR are found to be inversely proportional to pressure, associated with the mean free path between H_2_ molecules. Bulk diffusion predominant in the case of the mean free path (λ) is less than the pore diameter found in large pores or high-pressure gas diffusion. The bulk diffusion coefficient ($D_{B})$ can be expressed as [35];

(10) $$D_{B}=\frac{1}{3}\lambda \upsilon =\frac{1}{3}\frac{5}{8}\frac{\mu }{P}\sqrt{\frac{RT\pi }{2M}}\upsilon$$

where μ is the viscosity of the diffusion molecule in units of kg m/s and P is the pressure. The factor 5/8 considers the Maxwell–Boltzmann distribution of molecular velocity. The experimental results of the diffusion coefficient in Figure 6 are fitted by both Equations (9) and (10), as indicated by the blue and black lines, respectively. In the region of Knudsen diffusion below critical pressure, the diffusion coefficient is proportional to the pressure, which may be caused by an increase in the porosity in Equation (9) due to an increase of the pressure. The decrease in the bulk diffusion coefficient is attributed to a decrease in the mean free path with increasing pressure. Meanwhile, Figure 6 shows that the arrow position shifts to the high-pressure side with increasing filler content. The findings may be caused by a decrease in the peak height at the arrow position, which is responsible for the decreased diffusivity by adding the fillers. We are further seeking the origin for the interesting results.

Similar to Figure 4a,c, the deviation of diffusivity from bulk diffusion fit above 60 MPa for NBR H60 and for NBR S60 indicates abrupt fast hydrogen diffusion, which may also be caused by the formation of a hydrogen path channel at higher pressure. This observation will be discussed with the SEM image in the following section.

**Figure 7:**
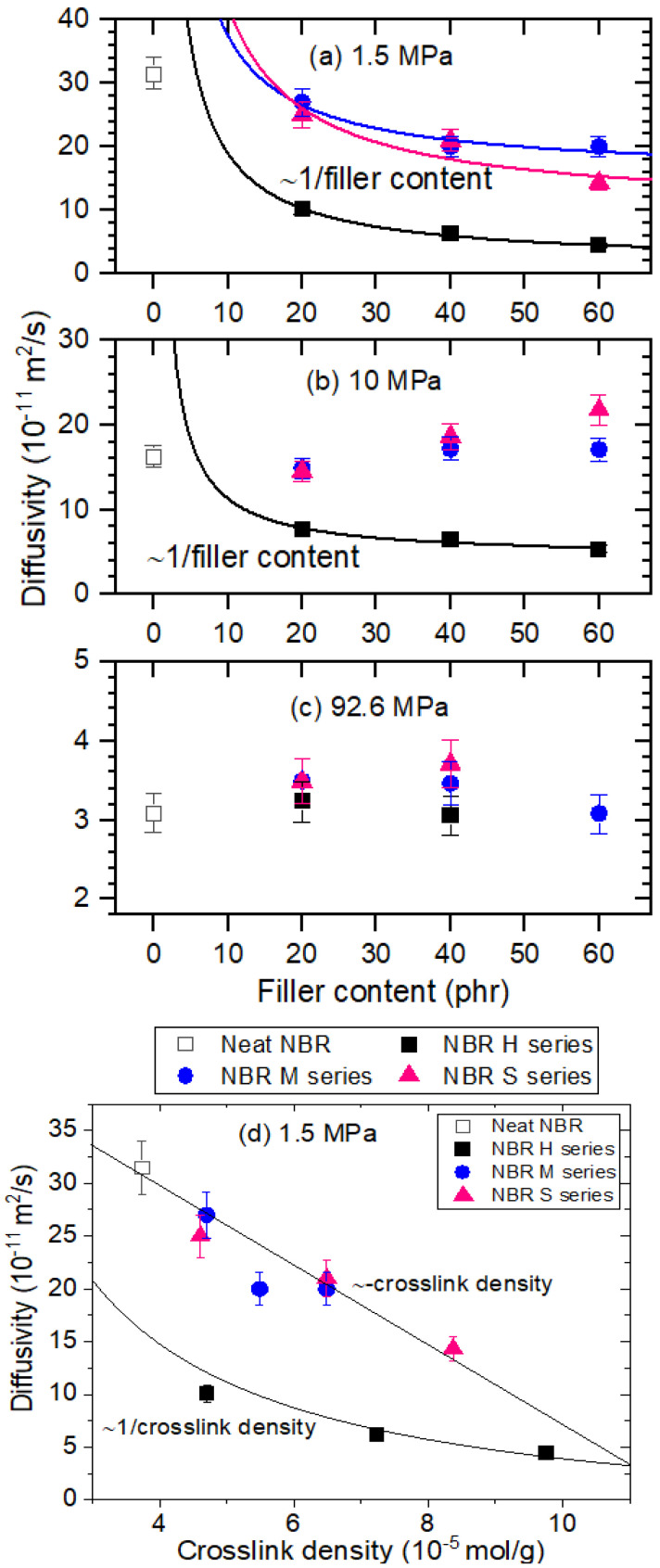
Diffusivity versus different filler content at exposed pressures of (**a**) 1.5 MPa, (**b**) 10 MPa, (**c**) 92.6 MPa and (**d**) diffusivity versus crosslink density in NBR composites blended with CB and silica. The lines in (**a**,**b**) are fitted with the relationship between diffusivity and reciprocal filler content. The line in (**d**) for the NBR H series is fitted with the relationship between diffusivity and reciprocal crosslink density. The line in (**d**) for the NBR M and S series is fitted with a negative linear relationship between diffusivity and crosslink density.

Figure 7a–c shows the variation of diffusivity versus filler content at three different pressures of 1.5 MPa, 10 MPa and 92.6 MPa, respectively. At low pressure of 1.5 MPa, all fillers suppress diffusion due to increased tortuosity by the filler, resulting in a decrease in the diffusion rate of ~1/filler content, indicated in the form of an asymptotic line. At low pressure of 1.5 MPa, the decrease in the diffusivity with the filler content for HAF-filled NBR is larger than the decrease in the diffusivity for MT CB and silica-filled NBR, possibly related to the filler-polymer interaction, mobility change of polymer chain by the volume fraction of the filling agents and activation energy for diffusion rate, reported in other literature [36,37,38].

However, with increasing pressure up to 92.6 MPa, the filler effect on diffusion is reduced, and the diffusivity for all specimens converges at a value of approximately 3 × 10^−11^ m^2^/s, except for those for NBR H60 and NBR S60 due to hydrogen channel formation. Figure 7d displays the correlation between diffusivity versus crosslink density of specimens. The diffusivity for the NBR H series is related to the reciprocal crosslink density, which exhibits a trend similar to the diffusivity versus filler content at low pressure, whereas the diffusivity for the NBR M series and S series decreases linearly with increasing crosslink density. From the two fitted lines of Figure 7d, the diffusivity value for all filled NBR is shown to converge to the same value at a crosslinked density of 9 × 10^−5^ mol/g, which could control the diffusion from the permeation properties.

The crosslink density (ν) for the EPDM polymer composites is calculated by Flory–Rehner Equations as [39,40,41]:

(11) $$\nu =\frac{1}{2M_{c}}=-\frac{\ln\left(1-V_{1}\right)+V_{1}+\chi V_{1}^{2}\;}{2\rho_{r}V_{0}\left(V_{1}^{\frac{1}{3}}-\frac{V_{1}}{2}\right)}$$

(12) $$V_{1}=\frac{\frac{W_{d}-W_{f}}{\rho_{r}}}{\frac{W_{d}-W_{f}}{\rho_{r}}+\frac{W_{s}-W_{d}}{\rho_{s}}}$$

where $\;M_{c}$ is the average molecular weight between crosslinks, $V_{0}$ is the molar volume of the solvent (cm^3^/mol) and $V_{1}$ is the volume fraction of rubber in the swollen network at equilibrium. $W_{d}$ is the weight of the unswollen sample, $W_{f}$ is the weight of the filler in the compound, and $W_{s}\;$is the weight of the swollen sample, $\rho_{r}\;$is the density of EPDM composites, and $\rho_{s}\;$is the density of the THF, and χ is the polymer–solvent interaction parameter (χ = 0.501).

**Figure 8:**
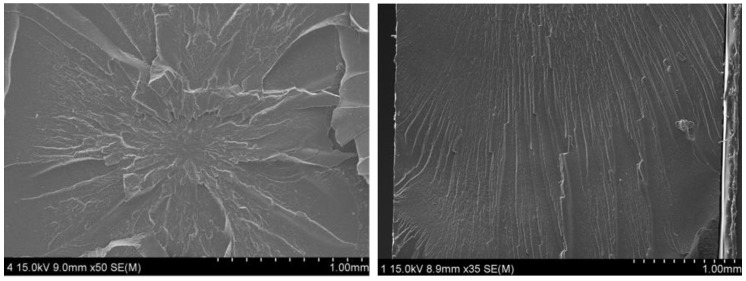
SEM images of NBR H60 (**left**) without and (**right**) with hydrogen exposure at a pressure of 60 MPa.

**Figure 9:**
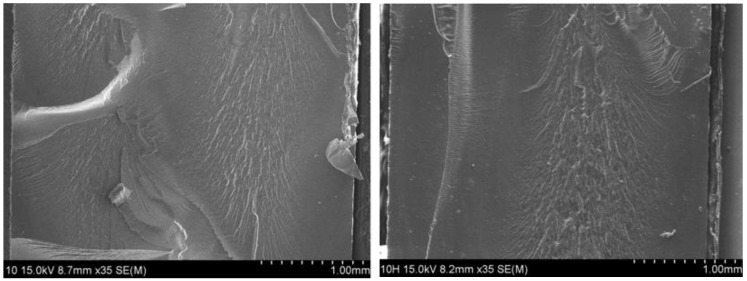
SEM images of NBR S60 (**left**) without and (**right**) with hydrogen exposure at a pressure of 60 MPa.

As shown in Figure 4 and Figure 6, the behavior for both NBR H60 and NBR S60 exhibits an abrupt increase in hydrogen uptake and diffusion above 60 MPa, which may be attributed to the diffusion channel by percolation. To determine the correlation between the formation of hydrogen channels and phenomenology, the SEM images in Figure 8 and Figure 9 were supplemented with NBR H 60 and NBR S 60, respectively, without and with exposure to 60 MPa H_2_. After exposure to H_2_, the morphology of NBR H60 shown on the right side of Figure 8 is changed from a random distribution on the left side to a uniaxially directed distribution. One possibility is that pore percolation occurs during the permeation of H_2_ gas under 60 MPa of ambient pressure stress and causes density modulation. one can know that the channeling structure occurred over the entire cross-sectional surface. Especially, this is true for the case of HAF CB filler (Figure 8). However, such a phenomenon is relatively weak in the case of silica filler (Figure 9). As described in our experimental section, our SEM images are obtained on the cryo-fractured surface. Meanwhile, if the SEM images are taken from the sample surface prepared by knife cutting, the observation of either microcracks or microvoids can also be possible.

**Figure 10:**
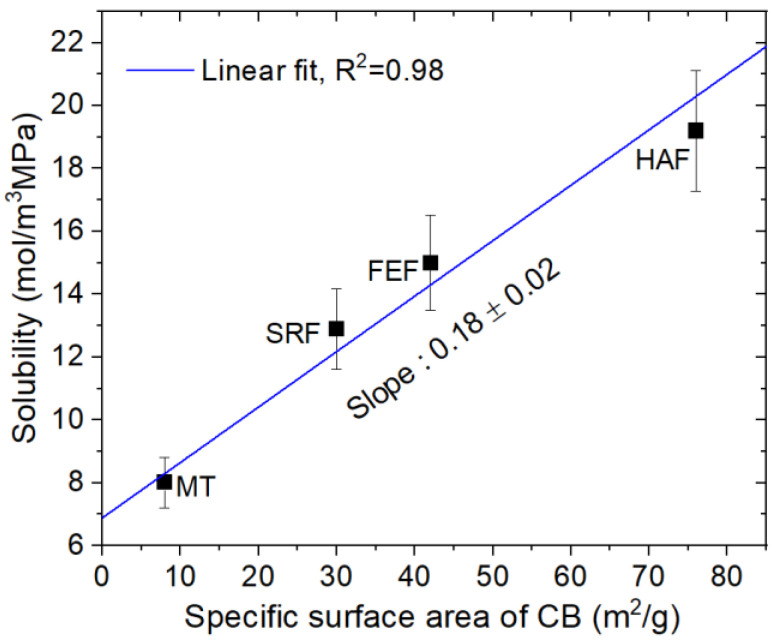
Linear correlation between solubility and specific surface area of CB particle.

In addition to the size or surface area of CB filler, we try to find the additional considered factors causing the different values in solubility and diffusivity of H_2_ for CB filled-NBR composites. We discovered the linear correlation (Figure 10) between solubility and surface area for CB particles in filled NBR composites, with a good squared correlation coefficient (R^2^ = 0.98). This may imply that the dominant contribution from solubility is mainly originated from the surface area of filler particles rather than any additional effects. However, because H_2_ sorption phenomena in two CB filled-NBR are greatly different from each other, that is, single sorption in MT CB and dual sorption in HAF CB depending on CB fillers type. Thus, the difference in the diffusivity between MT CB and HAF CB could not be attributed merely to the surface area of CB particles. Unlike MT CB filled-NBR composites, the attractive trapping of adsorbed H_2_ by the HAF CB filler surface and activation energy for diffusion, which is related to the structure of CB, could be a factor resulting in the slower diffusion rate than that in MT CB filled-NBR. However, to clarify the nature of this phenomenon, we are seeking the origin of different permeability between two CB filled-NBR composites. 

## 5. Conclusions

An effective volumetric analysis is applied to investigate hydrogen permeation with a diffusion analysis program. The filler effects on H_2_ pressure-dependent diffusion for NBR blended with CB and silica fillers are systematically investigated as a function of pressure, filler spices and filler concentration.

The pressure-dependent filler effect for all NBR composites displays particular characteristics depending on filler species and contents. NBR blended with MT CB and silica fillers exhibits a single sorption behavior following Henry’s law up to 92.6 MPa, and the solubility comes from hydrogen absorbed into the polymer network. However, dual sorption behaviors are observed for NBR blended with HAF CB fillers, satisfying both Henry’s law and the Langmuir model up to 92.6 MPa. The hydrogen solubility of HAF CB-filled NBR has two contributions: absorption in the neat polymer and adsorption at the HAF CB filler surface. The contribution from HAF CB filler is proportional to filler content. The difference in CB filler effects may be attributed to the specific surface area of the CB filler.

The pressure-dependent diffusivity is comprised of the contribution from Knudsen diffusion below 3~7 MPa and bulk diffusion above the pressures. The Knudsen diffusion coefficient is proportional to the pressure and is responsible for an increase in the porosity by increasing the pressure. The decrease in the bulk diffusion coefficient is related to a decrease in the mean free path with increasing pressure. The crossing position of the contribution of two diffusions shifts to the high-pressure side with increasing filler content, which may be attributed to decreased diffusivity by introducing the fillers. The filler effect on the diffusion rate is dependent on the exposed pressure. At low pressure of 1.5 MPa, the filler suppresses diffusion due to increased tortuosity by the filler. When increasing the pressure to 92.6 MPa, the filler effect on diffusion is weak, and the diffusivity for all specimens converges to a fixed value, except for those for NBR H60 and NBR S60 by hydrogen percolation channel formation, which is confirmed by SEM image observation.

Filler dependence on solubility and diffusivity is remarkably observed in the NBR H series compared to the NBR M and S series, which is related to the specific surface area of CB and the interfacial structure between CB and silica filler. A negative linear relationship between diffusivity and crosslinking density for NBR M and S is found, while a linear correlation between diffusivity and reciprocal crosslink density for the NBR H series is observed.

## Figures and Tables

**Table 1:** Chemical composition of the NBR series with HAF and MT CB fillers.

Chemical Composition	Neat NBR	NBR-H20	NBR-H40	NBR-H60	NBR-M20	NBR-M40	NBR-M60
KNB 35 L	100	100	100	100	100	100	100
ZnO	3.0	3.0	3.0	3.0	3.0	3.0	3.0
St/A*	1.0	1.0	1.0	1.0	1.0	1.0	1.0
HAF N330	-	20	40	60	-	-	-
MT N990	-	-	-	-	20	40	60
S	1.5	1.5	1.5	1.5	1.5	1.5	1.5
TBBS^+^	0.7	0.7	0.7	0.7	0.7	0.7	0.7

St/A*: Stearic Acid, TBBS^+^: N-Tert-Butyl-2-Benzothiazole Sulfenamide.

**Table 2:** Chemical composition of the NBR series with silica fillers.

Chemical Composition	NBR-S20	NBR-S40	NBR-S60
KBR 35 L	100	100	100
ZnO	3.0	3.0	3.0
St/A	1.0	1.0	1.0
Silica S-175	20	40	60
Si-69^×^	1.6	3.2	4.8
PEG^#^	0.8	1.6	2.4
S	1.5	1.5	1.5
TBBS^+^	0.7	0.7	0.7

Si-69^×^: Silane coupling agent, PEG^#^: Polyethylene glycol, TBBS^+^: N-tert-Butyl-2-Benzothiazole Sulfenamide.

## Data Availability

The data used to support the findings of this study are available from the corresponding author upon request.
